# Principles and Illustrations of Pathological Anatomy; Being a Complete Series of Colored Lithographic Drawings

**Published:** 1845-07

**Authors:** 


*Principles and Illustrations of Pathological Anatomy; being a complete series of Colored Lithographic Drawings.* By J. Hope, M. D., F. R. S., Physician to the St. Mary-Le-Bone Infirmary; Mem. Hon. De La Société De Statistique Universelle; Extraord. Mem. and formerly Pres. of the Roy. Med. Soc. Ed., etc. First American Edition. Edited by L. M. Lawson, M. D., Professor of General and Pathological Anatomy and Physiology in Transylvania University. Royal 8vo. Cincinnati: Desilver and Burr. Lexington: A. T. Skillman and Son. 1844.

Next in importance to a knowledge of the structure of the various organs and parts of the human body, in a state of health, must be regarded the changes and modifications that result from disease. Whether the morbid actions which constitute the various disorders that flesh is heir to, cause or result from these structural alterations, in the first instance, the facts are equally necessary to be known by those who would justly appreciate the symptoms of disease as indications for rational treatment. The study of symptoms, without an inquiry into the lesions they denote or portend, is but an idle task; nor is the attempt to frame a system of rational therapeutics, without a previous acquaintance with the uses and actions of the various parts of the body in health, and their aberrations in disease, at all less absurd. In the language of a distinguished living author: "The whole department of practical medicine teems with examples of the benefits which it has derived from *morbid anatomy.* What should we know of the nature, products, and tendencies of inflammations, and other diseases which alter the structure, but for the scalpel revealing them to our very sight and touch? The minuteness with which it (morbid anatomy) has been pursued in connexion with clinical observation, in regard to diseases of the lungs, heart, liver, kidneys, and alimentary canal, deserves especially to be mentioned as the great source of our improved theory and practice in these complaints."—(*Williams's Principles of Medicine.*)

Impressed with the importance of this subject, we rejoice at every effort to facilitate and encourage its prosecution. Simple description, even when accompanied by ample commentaries, without demonstration, in subjects so difficult to comprehend, is always dry and unsatisfactory. As a preparatory step in such investigations, well executed drawings, properly colored, are most essential aids to the beginner. It is in this light only, however, that we can commend them to our junior brethren: nothing can adequately substitute the actual inspection of the altered tissues themselves. It is to the cadaver, with scalpel in hand, and frequently with glasses, too, that we must go to acquire just ideas of the extent and character of the changes that result in the progress of disease.

Dr. Hope's work on pathological anatomy has been before the profession about a dozen years, and has received its marked approval;—it is, indeed, a work of very great merit, and worthy to be in the hands of every man who aspires to a competent knowledge of the healing art. The opportunity is now presented to American physicians to possess themselves of it at a very reasonable cost. In regard to the execution of the present edition, which is the first that has appeared in this country, we are free to say that the paper and typography are very respectable, and do credit to the press and the liberality of the publishers of the "Queen City of the West." Of the illustrations, in the copy we have seen, we are bound to confess, however, that they are not at all equal to the originals: in the coloring, especially, they are particularly deficient. This is to be ascribed to the infancy of the art in the West, and the almost impossibility of getting such a work properly executed. But we are pleased to learn that arrangements have been made in this city to have the coloring done in future by an experienced hand, and in a superior manner, so that copies which may hereafter be issued will approach the European edition in excellence. The remarks and observations of the Editor, although not numerous, are apposite and well expressed, and afford creditable evidence of judicious reading and reflection.

